# Investigation of enhanced hemocompatibility and tissue compatibility associated with multi-functional coating based on hyaluronic acid and Type IV collagen

**DOI:** 10.1093/rb/rbv030

**Published:** 2016-02-25

**Authors:** Jingan Li, Kun Zhang, Wenyong Ma, Feng Wu, Ping Yang, Zikun He, Nan Huang

**Affiliations:** ^1^Key Laboratory for Advanced Technologies of Materials, Ministry of Education, School of Material Science and Engineering, Southwest Jiaotong University, Chengdu 610031, People’s Republic of China;; ^2^School of Life Science, Zhengzhou University, 100 Science Road, Zhengzhou 450001, People’s Republic of China; ^3^Center of Stem Cell and Regenerative Medicine, First Affiliated Hospital of Zhengzhou University, 40 University Road, Zhengzhou 450052, People’s Republic of China

**Keywords:** cardiovascular devices, biocompatibility, surface modification, hyaluronan, Type IV collagen

## Abstract

The biocompatibility of cardiovascular devices has always been considered crucial for their clinical efficacy. Therefore, a biofunctional coating composed of Type IV collagen (CoIV) and hyaluronan (HA) was previously fabricated onto the titanium (Ti) substrate for the application of promoting vascular smooth muscle cell contractile phenotype and improving surface endothelialization. However, the anti-inflammation property, blood compatibility and *in vivo* tissue compatibility of the HA/CoIV coating, as paramount consideration of cardiovascular materials surface coating, have not been investigated. Thus, in this study, the three crucial properties of the HA/CoIV coating were tested. The platelet adhesion/activation test and the dynamic whole blood experiment implied that the HA/CoIV coating had better blood compatibility compared with Ti substrate and pure CoIV coating. The macrophage adhesion/activation and inflammatory cytokine release (tumor necrosis factor-alpha and interleukin-1) results indicated that the HA/CoIV coating could significantly improve the anti-inflammation property of the Ti substrate. The *in vivo* implantation of SD rats for 3 weeks’ results demonstrated that the HA/CoIV coating caused milder tissue response. All these results suggested that the multi-functional HA/CoIV coating possessed good biocompatibility. This research is anticipated to be potentially applied for the surface modification of cardiovascular stents.

## Introduction

Cardiovascular implant applied on the clinical treatment of cardiovascular disease (CVD) should perform its function with good blood compatibility, especially in the early stages after implantation [1]. The adsorption/conformational change of plasma proteins and the adhesion/activation of platelets on the device surfaces may induce thrombus formation and further implant failure [1]. Inhibition of inflammation is another consideration during the whole implantation process and posttreatment [2, 3]. The macrophages is one of the first cells to arrive at the tissue-implant-interface during the inflammation, and their attachment and activated by biomaterials can release chemotatic factors, adhesion molecules, chemokines and cytokines, such as tumor necrosis factor-alpha (TNF-α) and interleukin-1 (IL-1) [4]. During the implantation, macrophage aggregation and activation, inflammatory cytokines release may induce pathological growth and migration of vascular smooth muscle cells (SMC) and endothelial cells (EC) at the diseased part and/or injuried site of the vessel [5–7]. This process accompanied by disordered action of the clotting factor and the conformational change/agglomeration of fibrinogen may induce the events of in-stent thrombosis and intimal hyperplasia [8–10], and then long-term and more serious inflammation [11, 12], which is detrimental for the long-term interventional treatment of CVD [13, 14]. Tissue compatibility of the implanted devices should also be considered because the implantation may trigger undue host response, such as aggregation and proliferation of fibroblasts [15, 16], and further lead to the vascular wall thickening and luminal restenosis [17, 18]. Therefore, enhancing the blood compatibility, tissue compatibility and anti-inflammation of the implanted devices is overwhelmingly crucial for their clinical application.

Surface modification of biomaterials has been widely researched for the purpose of enhancing their biocompatibility [19–21]. Immobilizing endothelial extracellular matrix (ECM) components (collagen [22], fibronectin [23], laminin [24] and hyaluronan (HA)[25], etc.) and other biomolecules (heparin [26], gallic acid [27], dopamine [28] and chondroitin sulfate [29], etc.) on the biomaterials has been fully developed as a mature approach for surface modification of cardiovascular biomaterials. However, how to construct a multi-functional microenvironment which has outstanding biocompatibility using these biomolecules requires further investigation.

It has been reported the interaction with HA can make *in vitro* CoIV structure more ordered and biomimetic to the CoIV in the natrual vascular endothelial ECM [30]. In the previous work, we constructed a novel biocoating composed of hyaluronic acid (HA, 1 × 10^6^ Da) and Type IV collagen (CoIV) [31, 32]. HA is a glycosaminoglycan which is one of main components of the EC ECM and owns multi-functional advantages for the application of biomedical materials, such as anti-coagulation [33], anti-inflammation [34] and anti-hyperplasia [35], etc. CoIV is another main ingredient of the EC ECM and constructs the skeleton of the EC ECM [36]. CoIV in natural ECM can improve EC attachment, proliferation and migration [37], meanwhile promote SMC contractile phenotype [31]. The previous constructed HA/CoIV coating has been proven better ability of anti-hyperplasia and endothelialization compared with the pure HA coating or CoIV coating, respectively [31, 32]. However, the effects of this multi-functional HA/CoIV coating on anti-coagulation and inhibition of inflammation have not been evaluated, and its *in vivo* tissue compatibility has also not been investigated.

**Figure 1.**
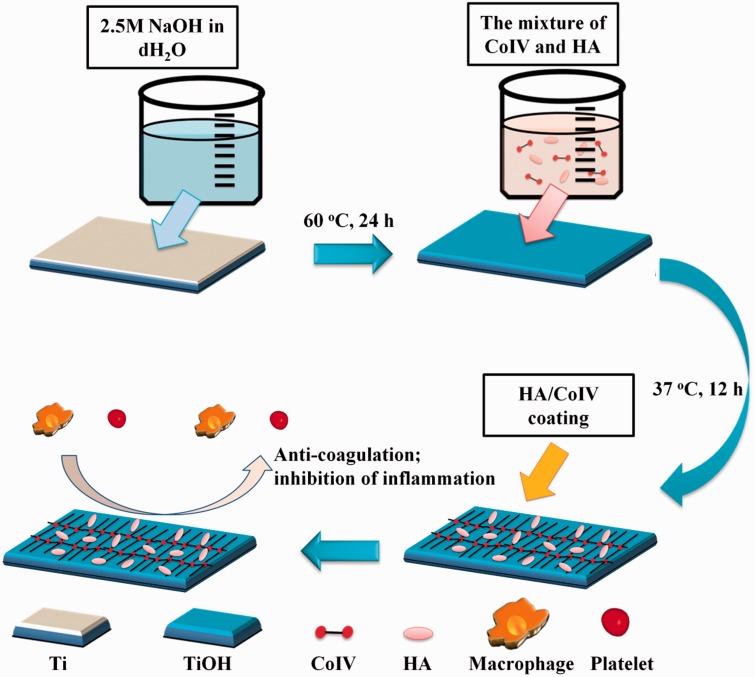
The scheme of preparing HA/PDA coating on Ti surface and its effect on anti-coagulation and anti-inflammation

In the present work, the blood compatibility (platelet adhesion/activation and the whole blood performance) and anti-inflammation property (macrophage adhesion/activation and inflammatory cytokines release) on the HA/CoIV coating were investigated, respectively (Fig. 1). In addition, subcutaneous implantation in SD rats was also investigated. We hope this HA/CoIV coating will not only be SMC and EC compatible but also blood and tissue compatible, which will be promising for the development of new generation of multi-functional and biocompatible cardiovascular devices.

## Materials and experiments

### Fabrication of the HA/CoIVcoating

The HA/CoIV coating was fabricated onto the 2.5 M NaOH treated titanium (Ti) substrate (labeled as TiOH) as our previous work described [31, 32]. Briefly, CoIV powder (Sigma, USA) was diluted to a concentration of 500 μg/ml with 5 mg/ml HA (Sangon Biological Engineering Co. Ltd, China) solution (deionized water) at pH 6.9. Then, the TiOH sample was immersed in this mixture and incubated for 12 h at 37°C. After the rinsed step (deionized water, three times, 5 min each time), the sample was dried at room temperature. TiOH samples immersed in 500 μg/ml CoIV solution (diluted in acetate buffer, pH 4) (labeled as CoIV/TiOH) and 5 mg/ml HA (labeled as HA/TiOH) incubated for 12 h at 37°C were used as control.

### Fluorescence staining of CoIV on the HA/CoIVcoating

The fluorescence staining of CoIV on the HA/CoIV coating and CoIV/TiOH sample were performed using the related kits to observe the structure difference. This characterization was also used to confirm the successful preparation of the HA/CoIV coating on the TiOH surface.

### Blood compatibility of the HA/CoIVcoating

The blood compatibility of the HA/CoIV coating was investigated by the platelet adhesion and the whole blood test, respectively, and the Ti, TiOH, CoIV/TiOH and HA/TiOH samples were used as control.

For the platelets adhesion test, fresh whole blood of a volunteer was centrifuged at 1500 rpm for 15 min to obtain the platelet-rich plasma (PRP), then the PRP was added to the samples surfaces and incubated at 37°C for 1 h. After washed with normal saline (NS, pH = 7.4) for three times (each time 5 min), the adherent platelets were fixed with 4% paraformaldehyde at 4°C for 2 h. The morphology of the adhered platelets was examined by a Rhodamine123 staining (Sigma, USA) and observed under a fluorescence microscope (DMRX, Leica, Germany) [38]. The amounts of the adherent platelets and the activated platelets were determined by the Lactate dehydrogenase (LDH) and GMP140 assay, respectively [39, 40].

For the whole blood test, the HA/CoIV and the control samples were set into a flow chamber device *in vitro*, and the whole blood flow with 15 dyn/cm^2^ speed was applied in the devices at 37°C for 1 h. Then, the samples were taken out, washed with NS (pH = 7.4) for three times (each time 5 min) and fixed with 2.5% glutaraldehyde solution. After the rinsed and further dried step, the samples were coated with gold to conduct SEM to evaluate the morphology [41].

### Anti-inflammation property of the HA/CoIVcoating

The amounts of the attached macrophages and their inflammatory cytokines were detected as the evaluation of the anti-inflammation property of the HA/CoIV coating in this study. The peritoneal macrophages extracted from the SD rats were obtained as the previous work described [33] and cultured at 37°C in a humidified atmosphere containing 95% air and 5% CO_2_. Replicated cultures were performed when cells approached confluence, and cells were fed with fresh prepared growth medium every 48 h. The cells were added onto each samples with the concentration of 5 × 10^4^ cells/ml and incubated at 37°C for 24 h. To study the extent of cell morphology and spreading, a Rhodanmine123 staining was used for the attached macrophages, and the numbers of the cells on each samples were statistically counted from at least 15 random pictures [42]. The supernatant harvested after macrophages cultured on samples for 24 h was applied as the specimen, and the TNF-α and IL-1 release were measured using related kits according to the manual [4]. All the inflammatory cytokines were finally normalized to the cell number.

### *In vivo* tissue response test of HA/CoIVcoatings

In this study, three adult SD rats weighing ∼0.4 kg were used. The double mirror-polished bare Ti disks (diameter = 10 mm, *n* = 3), TiOH disks (diameter = 10 mm, *n* = 3), HA coated TiOH disks (diameter = 10 mm, *n* = 3), CoIV coated TiOH disks (diameter = 10 mm, *n* = 3) and HA/CoIV coated disks (diameter = 10 mm, *n* = 3) were subcutaneously implanted on the back of both sides. After 3 weeks, the tissue around the implanted samples was harvested to evaluate the *in situ* body response reaction. The harvested tissue was fixed in 10% formaldehyde for 4–5 days. After washing by NS, the harvested tissue was then dehydrated in graded ethanol, saturated with xylene and embedded in paraffin. Hematoxylin and eosin (HE) staining of paraffin sections were performed for further histological analysis [15].

### Statistical analysis

The data were statistically evaluated using analysis of variance (ANOVA) by homogeneity test of variances first, and *post hoc* test was prepared subsequently in Fisher's Least Significant Difference (statistics; analysis) (LSD) method for comparison. They were expressed as mean ±  SD. The probability value *P* < 0.05 was considered as a significant difference. The data analysis was performed using the software SPSS 11.5 (Chicago, IL).

## Results and discussion

### Quality characterization of the HA/CoIVcoating

**Figure 2.**
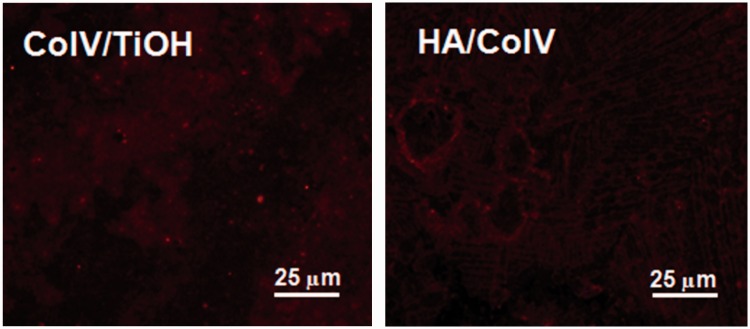
Immunofluorescence staining images of CoIV on the samples of CoIV/TiOH and HA/CoIV

Figure 2 depicts the immunofluorescence images of CoIV on CoIV/TiOH and HA/CoIV samples. The CoIV exhibited distribution in dispersed clusters on CoIV/TiOH surface, whereas showed mesh-like distribution with microscopically visible fibers on the CoIV/HA coated surfaces, and the diameter of the mesh ranged from 20 to 30 µm which might contributed to the migration of EC. On the HA/CoIV coating, several oriented microstripes could also be seen clearly, and this structure was able to induce EC biomimetic elongation to release more anti-coagulant factor, such as nitric oxide, prostacyclin and thrombomodulin [32]. The results above not only indicated the successful preparation of the HA/CoIV coating on the TiOH surface but also proved that the mixture of CoIV and HA made the CoIV distribution more ordered and biomimetic.

### Hemocompatibility of the HA/CoIVcoating

**Figure 3.**
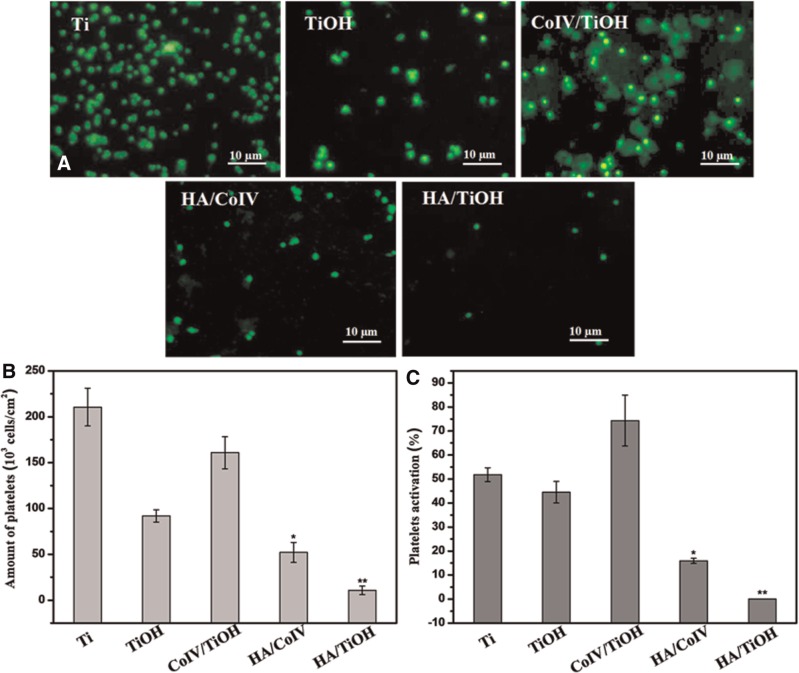
(**A**) Fluorescence images of platelets on the samples of Ti, TiOH, CoIV/TiOH, HA/CoIV and HA/TiOH; amounts of (**B**) attached and (**C**) activated platelets on the samples of Ti, TiOH, CoIV/TiOH, HA/CoIV and HA/TiOH (*P < 0.05 compared with Ti, TiOH and CoIV/TiOH, **P < 0.05 compared with Ti, TiOH, CoIV/TiOH and HA/TiOH, mean ± SD, *n* = 3)

The *in vitro* platelet adhesion test was used to investigate the hemocompatibility of the HA/CoIV coating preliminarily. The representative fluorescence images of platelets adhesion behavior on these surfaces are depicted in Fig. 3A. Platelets attached and aggregated markedly on Ti substrate, TiOH sample and CoIV/TiOH sample with marked shape change (spreading) and pseudopod formation, especially the platelets attached on the CoIV/TiOH, exhibited complete spreading morphologies, indicating serious activation. However, adhesion and aggregation of the platelets on HA/CoIV coating and HA/TiOH sample were significantly reduced for the presence of HA, and platelets on the HA conjugated sample maintained their round shapes with no formation of pseudopods. The quantity of platelets on different samples detected by the LDH method was presented in Fig. 3B. The data showed that the Ti substrate, TiOH sample and CoIV/TiOH sample facilitated a much higher level of platelet adhesion compared with samples of HA/CoIV coatings and HA/TiOH (*P *<* *0.05). The quantity of adhered platelets on all the samples increased in the order: HA/TiOH< HA/CoIV coating < TiOH < CoIV/TiOH < Ti. The activated ratio of the adherent platelet on different samples examined by the GMP140 assay was displayed in Fig. 3C. The quantity of activated platelets was in consistent with the fluorescence images, and increased in the order: HA/TiOH< HA/CoIV coating < Ti and TiOH < CoIV/TiOH. It was clear that the platelets on the CoIV/TiOH possessed the biggest activated ratio, while it was interesting that the more CoIV on the HA/CoIV coating (description in the previous work [31, 32]) didn’t make more activation. More HA amount on the HA/CoIV coating provided the most contribution [31, 32] but the ordered CoIV distribution might be another important reason [33].

**Figure 4.**
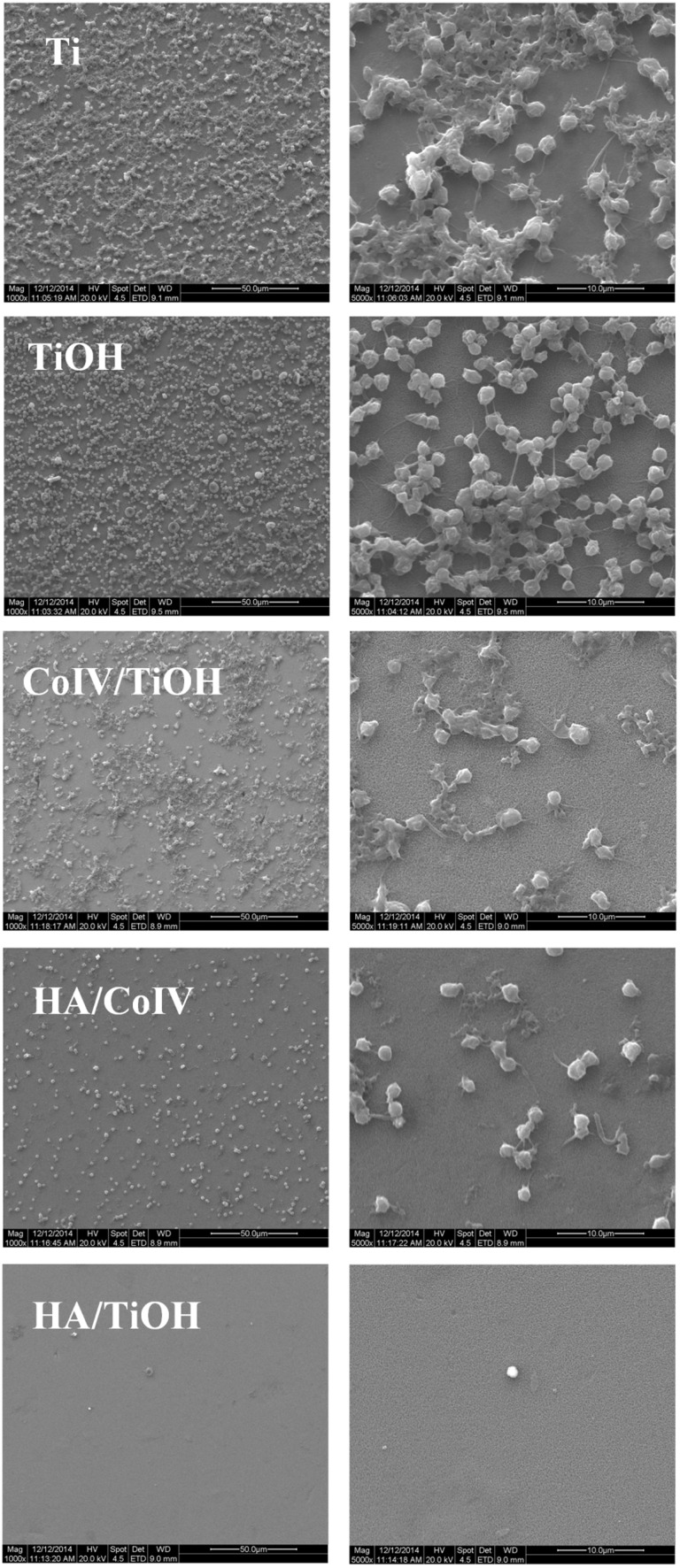
Typical SEM photograph of the whole blood ingredient attached on the samples of Ti, TiOH, CoIV/TiOH, HA/CoIV and HA/TiOH under a 15 dyn/cm^2^ BFSS for 1 h

To investigate the overall hemocompatibility of the HA/CoIV coating in further, the whole blood test under a biomimetic blood flow (BFSS: 15 dyn/cm^2^) was performed, and the representative typical SEM images were depicted in Fig. 4. It was remarkable that there were lots of fibers, red blood cells (RBC) and activated platelets attached on the bare Ti, TiOH and CoIV/TiOH samples. The platelets gathered, outstretched their pseudopod and intertwined with a large number of fibers. However, the attachment of fibers, platelets and RBC on HA/CoIV and HA/TiOH samples were significantly reduced. All these results indicated that the HA/CoIV coating possessed better hemocompatibility compared with the bare Ti, TiOH and CoIV/TiOH samples but not enough compared with the HA/TiOH sample.

### Anti-inflammation property of the HA/CoIVcoating

**Figure 5.**
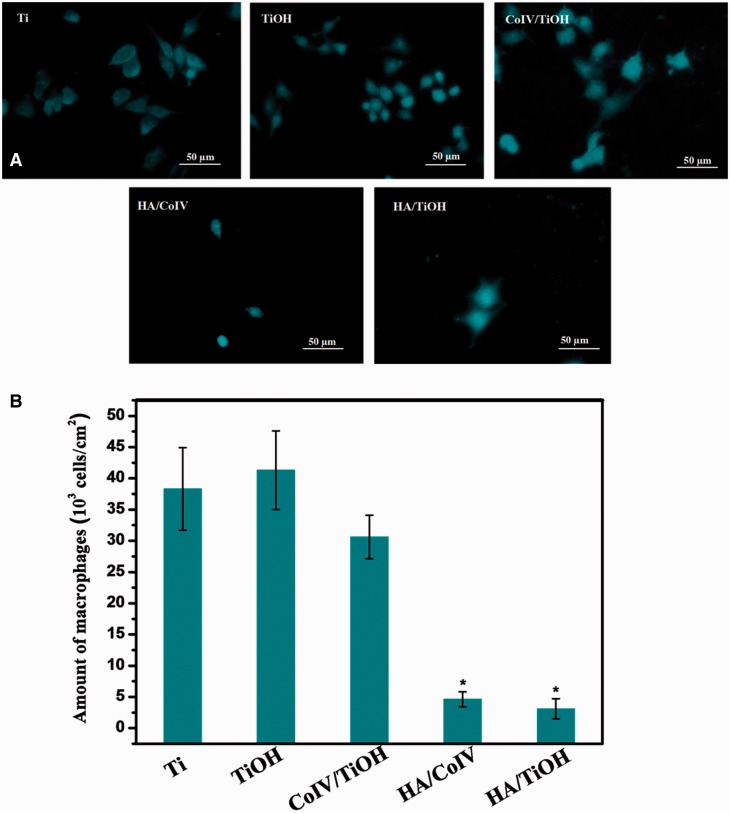
(**A**) Fluorescence images and (**B**) counts of attached macrophages on the samples of Ti, TiOH, CoIV/TiOH, HA/CoIV and HA/TiOH after incubation for 24 h (*P <* *0.05 compared with other samples, mean ± SD, *n* = 3)

**Figure 6.**
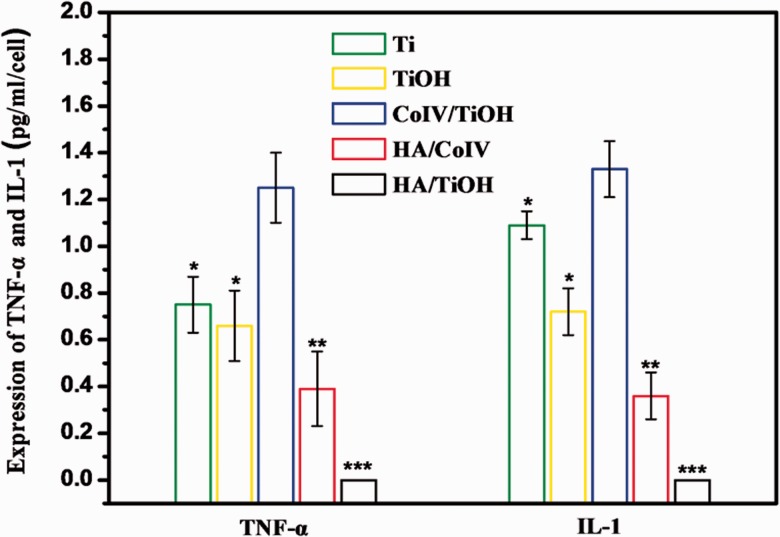
The amounts of (**A**) TNF-α and (**B**) IL-1 released from attached macrophageson the samples of Ti, TiOH, CoIV/TiOH, HA/CoIV and HA/TiOH after incubation for 24 h (*P <* *0.05 compared with CoIV/TiOH, **P <* *0.05 compared with Ti, TiOH and CoIV/TiOH, ***P <* *0.05 compared with the other samples, mean ± SD, *n* = 3)

For the biomedical implanted devices, it is an important criterion of macrophage adhesion, activation and inflammatory cytokine release for judging the degree of inflammatory response [4]. Thus, the attachment, activation and inflammatory cytokine release (TNF-α and IL-1) of the macrophages were performed to investigate the anti-inflammation property of the HA/CoIV coating. Obviously, the macrophages on the HA/CoIV coating presented round and shrinked morphology, suggesting non-activated phenotype, while the macrophages on the Ti, TiOH, CoIV/TiOH and HA/TiOH samples polygonal and spreading morphology, and this indicated the activated phenotype (Fig. 5A). The attached and activated macrophages on the HA/TiOH surface may be attributed to the exposure of the TiOH substate, which indicated the inhomogeneous distribution of the HA on the HA/TiOH surface. From the statistical count results of macrophages on each sample in Fig. 5B, the HA/CoIV coating and HA/TiOH sample exhibited significantly less adherent macrophages compared with Ti, TiOH and CoIV/TiOH samples. These results indicated that the HA/CoIV coating could inhibit macrophage adhesion and activation effectively. Figure 6 presents the expression of TNF-α and IL-1 released by macrophages after 24 h incubation on various samples. It was clear that the CoIV/TiOH surface showed more expression of TNF-α and IL-1, which indicated more activation of the macrophages, while the HA/TiOH surface showed almost no expression of the inflammatory cytokines, and the reason might be that there were too few macrophages attached on the HA/TiOH surface, thus the cytokines could not be detected. It had been reported that HA could effectively suppress adhesion, activation and inflammatory cytokines of the macrophages, thus possessed excellent anti-inflammation function [43]. It was notable that the HA/CoIV coating presented significantly less expression of TNF-α and IL-1 compared with CoIV/TiOH, Ti and TiOH samples attributing to the more HA immoblized on the coating. All the result indicated that the HA/CoIV coating could effectively enhance the anti-inflammation property of the surface.

### *In vivo* tissue response test of HA/CoIV coating

**Figure 7.**
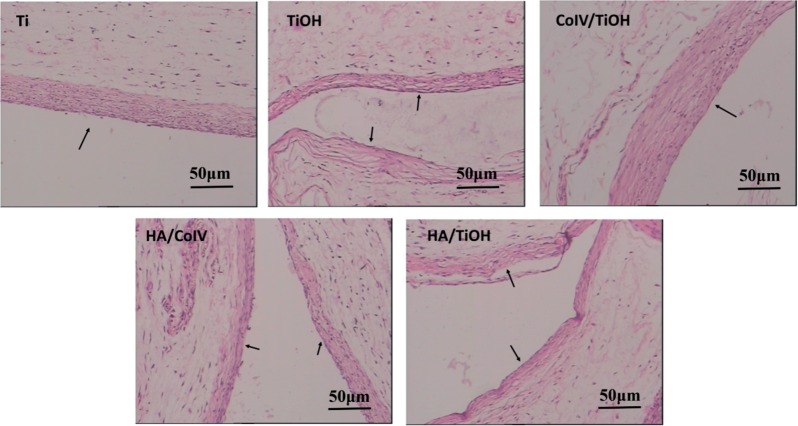
HE staining for subcutaneous tissues around Ti, TiOH, CoIV/TiOH, HA/CoIV and HA/TiOH after 3 weeks, respectively

When a biomedical device is implanted inside the patient, good tissue compatibility is required, because the local tissue response may influences its safety and performance *in vivo* [44, 45]. To fully rate the tissue response to HA/CoIV coating, samples were implanted subcutaneously implantation in SD rats. After 3 weeks, the tissue around the implant site was harvested for *in situ* analysis. HE (Fig. 7) of sections were performed for histological analysis. Neither bare Ti nor the HA/CoIV samples induced local toxic reactions. There were less inflammatory cell infiltration and granulation tissue development presented on HA/CoIV sample and HA/TiOH sample. The introduction of the HA/CoIV altered the tissue response but did not completely suppress fibrous capsule formation. For the Ti and TiOH substrates, the thickness of fibrous encapsulation surrounding the implanted site was 46.6 ± 3.6 μm and 21.5 ± 5.5 μm, respectively, at 3 weeks, while the thickness reduced to 20.8 ± 4.7 μm after coated with HA/CoIV, or 25.4 ± 4.4 μm after immobilized with HA. Although for the CoIV coated TiOH, the thickness significantly increased to 76.7 ± 1.6 μm, suggesting a more serious tissue response. It was included that the HA/CoIV coating showed acceptable and attenuated tissue response compared with the other samples, with less inflammatory cell infiltration, granulation tissue formation and thinner fibrous capsule development.

## Conclusion

In the present work, the micromesh structure composed of HA and CoIV (HA/CoIV coating) was successfully prepared onto the TiOH surface. Platelet adhesion/activation and the dynamic whole blood test indicated that the HA/CoIV coating possessed better blood compatibility compared with Ti, TiOH and CoIV/TiOH surfaces. Macrophage adhesion/activation and TNF-α/IL-1 release results proved that the HA/CoIV coating could effectively suppress inflammation. Additionally, *in vivo* experiment demonstrated that the HA/CoIV coating made milder tissue response suggesting excellent tissue compatibility. Nevertheless, the drug loading and delivery ability of this HA/CoIV layer should still be investigated. Thus, in the future work, we will choose one or more functional drug (such as heparin or metal nano-particle) to load in the HA/CoIV coating to endow the coating better biocompatibility.
